# Preface for the Special Issue “Cardiovascular Risk Factors and Socioeconomic Status in Japan: NIPPON DATA2010”

**DOI:** 10.2188/jea.JE20170242

**Published:** 2018-03-05

**Authors:** Katsuyuki Miura, Akira Okayama

**Affiliations:** 1 Co-Principal Investigators, the NIPPON DATA2010 Research Group; 2 Center for Epidemiologic Research in Asia, Shiga University of Medical Science, Shiga, Japan; 3 Department of Public Health, Shiga University of Medical Science, Shiga, Japan; 4 Research Institute of Strategy for Prevention, Tokyo, Japan

The National Integrated Project for Prospective Observation of Non-communicable Disease and its Trends in the Aged (NIPPON DATA) is a series of cohort studies on representative Japanese populations that participated in national surveys in Japan. These studies are unique because their participants are general Japanese adult populations from 300 randomly selected areas throughout Japan. NIPPON DATA80 and NIPPON DATA90 are cohort studies of the National Survey of Circulatory Disorders and the National Nutrition Survey in 1980 and 1990. These long-term cohort studies have provided a large amount of important evidence on the risk factors for non-communicable diseases, particularly cardiovascular diseases, and this information has been used in public health policy making, clinical guidelines, and health education in Japan.

A new cohort study, NIPPON DATA2010, was initiated in 2010.^1^ This is a cohort study of participants in the National Health and Nutrition Survey in 2010. NIPPON DATA2010 also includes original examinations and questionnaires added by our research group, as the successor of the National Survey of Circulatory Disorders. Study participants had also participated in the Comprehensive Survey of Living Conditions in 2010; therefore, the data of this national survey were merged with the NIPPON DATA2010 dataset. Thus, the dataset of NIPPON DATA2010 provides a range of information on the socioeconomic status of a representative Japanese population in 2010.

In this special issue of the *Journal of Epidemiology*, 11 studies on the relationships among socioeconomic status and obesity, dietary habits, and health-related behavior and knowledge, which are related to cardiovascular risk factors, are published, including the study profile paper of NIPPON DATA2010. Reductions in heath disparities in Japan are one of the main goals of the National Health Promotion Movement in the Twenty-first Century (“Health Japan 21”) (the second term).^2^ We hope that the information discussed in this issue will lead to important and effective measures that reduce health disparities and prevent cardiovascular diseases in Japan and worldwide.

We thank Drs. Nobuo Nishi, Nagako Okuda, Takayoshi Ohkubo, and Aya Kadota for their important contribution in editing this issue, and Dr. Keitaro Matsuo, the Editor-in-Chief of the *Journal of Epidemiology*, for his useful instructions and support.
